# The prognostic significance of the postoperative prognostic nutritional index in patients with colorectal cancer

**DOI:** 10.1186/s12885-015-1537-x

**Published:** 2015-07-16

**Authors:** Masatsune Shibutani, Kiyoshi Maeda, Hisashi Nagahara, Hiroshi Ohtani, Yasuhito Iseki, Tetsuro Ikeya, Kenji Sugano, Kosei Hirakawa

**Affiliations:** Department of Surgical Oncology, Osaka City University Graduate School of Medicine, 1-4–3 Asahi-machi, Abeno–Ku, Osaka City, Osaka Prefecture 545-8585 Japan

**Keywords:** Prognostic nutritional index, Colorectal cancer, Prognosis

## Abstract

**Background:**

The preoperative prognostic nutritional index (PNI) has been reported to correlate with the prognosis in patents with various carcinomas. However, the prognostic significance of the postoperative PNI is unknown. The aim of this study was to evaluate the prognostic significance of the postoperative PNI in patients with colorectal cancer (CRC).

**Methods:**

Two hundred and eighteen patients who underwent potentially curative surgery for stage II/III CRC were enrolled in this study. The PNI was calculated as 10 × serum albumin concentration (g/dl) + 0.005 × lymphocyte count (/mm^3^). The preoperative PNI was measured within two weeks before the operation and the postoperative PNI were measured at the first visit after leaving the hospital. We then examined the correlations between the preoperative/postoperative PNI and the prognosis for survival.

**Results:**

In the validation study, the median preoperative PNI was 47.90 (range: 32.45-61.36) and the median postoperative PNI was 48.69 (range: 32.62-66.96). According to the receiver operating characteristic (ROC) curve, we set 43.0 as the cut-off value in the validation study. For both the preoperative and postoperative PNI, the overall survival rates were significantly worse in the low PNI group in the validation study (preoperative PNI, *p* = 0.0374; postoperative PNI, *p* = 0.0005). In the multivariate analysis of the validation study, the combination of pre- and postoperative PNI was an independent predictor of poor overall survival (*p* = 0.006).

**Conclusions:**

The postoperative PNI is, in addition to the preoperative PNI, a useful prognostic marker. The combination of pre- and postoperative PNI was an independent prognostic factor in patients with CRC who underwent potentially curative surgery and is important for considering the long-term outcome in patients with CRC.

## Background

Colorectal cancer (CRC) is the third leading cause of cancer-related death worldwide [1]. Although the surgical procedures and chemotherapy have improved, a large number of patients relapse after curative resection, and the mortality from colorectal cancer is still high. Therefore, there has been a new focus on identifying biomarkers that can predict the prognosis. Although much attention has been paid to the factors related to the tumor in previous reports, increasing attention has recently been paid to the factors related to the host [2]. Among them, the prognostic nutritional index (PNI), which indicates the nutritional and immunological status of the host, and which has been used to predict the risk of complications after gastrointestinal surgery [3, 4], has been reported to correlate with survival in various types of cancer [5–9]. However, most of these reports investigated the preoperative status, and there have been no reports on the relationship between the postoperative PNI and the long-outcome after potentially curative surgery for CRC. The aim of this retrospective study was to evaluate the prognostic significance of the postoperative PNI in patients with CRC.

## Methods

### Patients

We retrospectively reviewed a database of 263 patients who underwent potentially curative surgery for stage II/III CRC at the Department of Surgical Oncology of Osaka City University between 2005 and 2011. We excluded 10 patients with ulcerative colitis, nine patients who had received preoperative therapy and 26 patients with multiple malignancies within five years. Therefore, 218 patients remained and were analyzed in this study.

All patients were followed up regularly with physical and blood examinations and mandatory screening using colonoscopy and computed tomography until June 2014 or death. Among the total 218 of patients, sixty-nine patients developed recurrent disease and 31 patients died.

The resected specimens were pathologically classified according to the seventh edition of the Union for International Cancer Control TNM classification of malignant tumors [10].

All patients were divided into two groups: including the exploratory group, which consisted of 32 patients who underwent surgery in 2005; and the validation group, which consisted of 186 patients who underwent surgery between 2006 and 2011.

### PNI

The preoperative blood samples were obtained within two weeks before the operation and the postoperative blood samples were obtained at the first visit after leaving the hospital. The PNI was calculated as 10 × serum albumin concentration (g/dl) + 0.005 × lymphocyte count (/mm^3^).

### Statistical analysis

The data were tested for normality by the Kolmogorov- Smirnov test. The significance of the correlations between the preoperative/postoperative PNI and the clinicopathological characteristics was analyzed using the χ^2^ test. The duration of survival was calculated according to the Kaplan-Meier method. Differences in the survival curves were assessed with the log-rank test. A multivariate analysis was performed according to the Cox proportional hazard model. All statistical analyses were conducted using the SPSS software package for Windows (SPSS Japan, Tokyo, Japan). Statistical significance was set at a value of *p* <0.05.

### Ethical consideration

This research was conformed to the provisions of the Declaration of Helsinki in 1995. All patients were informed of the investigational nature of this study and provided written informed consent. This retrospective study was approved by the ethics committee of Osaka City University.

## Results

### Clinical characteristics in the exploratory study

The patient characteristics are listed in Table 1. The patient population consisted of 20 males and 12 females, with a median age of 69 years (range: 42 to 86). Sixteen patients had tumors located in the colon and 16 had tumors located in the rectum. Among the total 32 of patients, fifteen patients received adjuvant chemotherapy. All of these patients received monotherapy using an oral prodrug based on 5-FU.

**Table 1:** The patient characteristics

	Exploratory Group (*n* = 32)	Validation Group (*n* = 186)
Gender		
Male	20	100
Female	12	86
Age (years)		
Median (range)	69 (42–86)	67 (26–86)
Location of primary tumor		
Colon	16	94
Rectum	16	92
Tumor depth		
≤T3	16	130
T4	16	56
Histological type		
Well, Moderately	27	172
Poorly, Mucinous	5	14
The number of lymph node metastases		
0	17	67
1-3	9	83
≥4	6	36
Preoperative CEA (ng/ml)		
≤5	14	126
>5	16	43
Preoperative CA19-9 (U/ml)		
≤37	13	155
>37	6	11
The amount of blood lost (ml)		
Median (range)	245 (5–1780)	97.5 (5–2700)
Length of operation (min)		
Median (range)	182 (76–437)	206 (93–687)
Complication(s)		
No	24	123
Yes	8	62
Regimen of adjuvant chemotherapy		
Monotherapy using an oral pro-drug based on 5-FU	15	126
Combination therapy with 5-FU and oxaliplatin	0	15
None	17	45
Preoperative PNI		
Median (range)	44.67 (31.76-60.24)	47.90 (32.45-61.36)
Postoperative PNI		
Median (range)	50.16 (31.89-60.75)	48.69 (32.62-66.96)
The number of hospitalization days		
Median (Interquartile range)	30 (25–39)	17 (13–24)
The number of postoperative days before the initiation of dietary intake (days)		
Median (Interquartile range)	4 (3–6)	3 (2–4)
The number of days from operation to the first hospital visit		
Median (Interquartile range)	31 (26–36)	28 (22–35)

*CEA* carcinoembryonic antigen, *CA19-9* carbohydrate antigen 19–9, *PNI* prognostic nutritional index

### Survival analysis according to the pre-/postoperative PNI in the exploratory study

**Fig. 1:**
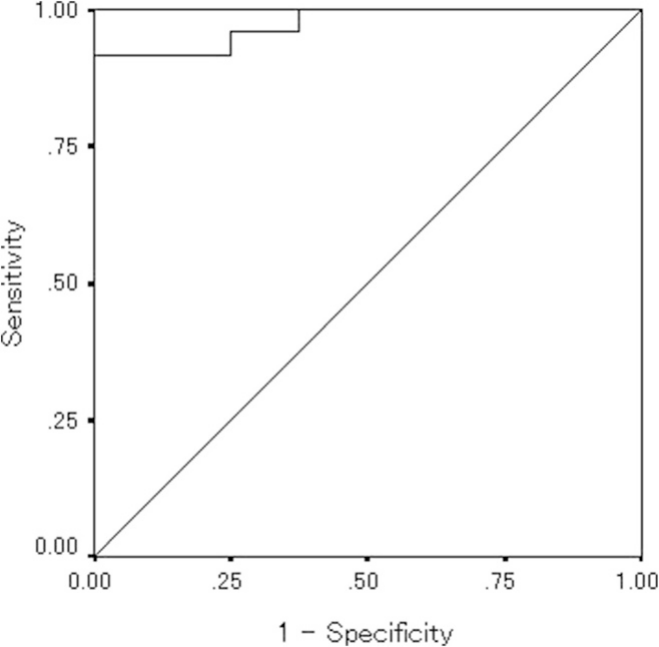
Receiver operating characteristic curve analysis of the postoperative PNI in the exploratory study. Area under the curve = 0.974, 95 % Confidence interval = 0.927-1.021, *p* < 0.001

**Fig. 2:**
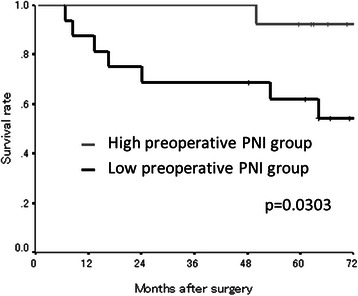
The Kaplan-Meier survival curves according to the preoperative PNI in the exploratory study. The overall survival rates were significantly worse in the low preoperative PNI group (*p* = 0.0303)

**Fig. 3:**
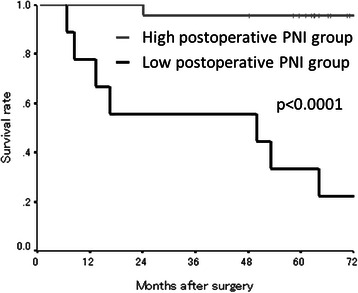
The Kaplan-Meier survival curves according to the postoperative PNI in the exploratory study. The overall survival rates were significantly worse in the low postoperative PNI group (*p* < 0.0001)

The median preoperative PNI was 44.67 (range: 31.76-60.24) and the median postoperative PNI was 50.16 (range: 31.89-60.75) (Table 1). The PNI distribution was normal. According to the receiver operating characteristic (ROC) curve, we set 45.0 as the cut-off value (the sensitivity was 91.7 % and the specificity was 87.5 %) (Fig. 1). Based on the cut-off value of 45.0, 13 patients were classified into the high preoperative PNI group and 16 patients were classified into the low preoperative PNI group. Moreover, 23 patients were classified into the high postoperative PNI group and 9 patients were classified into the low postoperative PNI group. With regard to the preoperative PNI, the overall survival rates were significantly worse in the low PNI group compared to the high PNI group (*p* = 0.0303) (Fig. 2). Moreover, the overall survival rates were also significantly worse in the low postoperative PNI group (*p* < 0.0001) (Fig. 3).

### Survival analysis according to the combination of the preoperative and postoperative PNI in the exploratory study

**Fig. 4:**
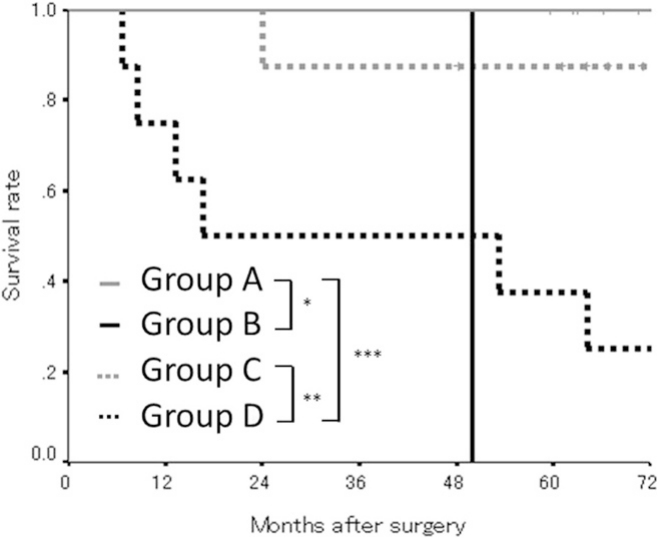
The overall survival subdivided according to the preoperative and postoperative PNI in exploratory study. The patients in group A exhibited a better prognosis than those in groups B and D (*, *p* = 0.0005; ***, *p* = 0.0003). The patients in group C exhibited a better prognosis than those in group D (**, *p* = 0.0163)

We categorized the patients into four groups according to the combination of their preoperative and postoperative PNI. The patients with high preoperative and postoperative PNI were categorized into group A. The patients with a high preoperative PNI and a low postoperative PNI were categorized into group B. The patients with a low preoperative PNI and a high postoperative PNI were categorized into group C, and the patients with a low preoperative and a low postoperative PNI were categorized into group D. The patients in group A exhibited a better prognosis than those in groups B and D (A vs. B, *p* = 0.0005; A vs. D, *p* = 0.0003). The patients in group C exhibited a better prognosis than those in group D (C vs. D, *p* = 0.0163) (Fig. 4).

### The correlations between the preoperative/postoperative PNI and the clinicopathological factors in the exploratory study

The preoperative PNI had a significant relationship with age (*p* = 0.008) and the number of hospitalization days (*p* = 0.027) (Table 2). The postoperative PNI had no significant relationships with any factors.

**Table 2:** The relationship between the clinicopathological factors and the preoperative/preoperative PNI in the exploratory study

	Preoperative PNI			Postoperative PNI		
	High	Low	*p*-value	High	Low	*p*-value
Gender						
Male	10	9		14	6	
Female	3	7	0.433	9	3	1.000
Age (years)						
≤70	11	5		13	4	
>70	2	11	0.008	10	5	0.699
Location of primary tumor						
Colon	7	9		13	5	
Rectum	6	7	1.000	10	4	1.000
Tumor depth						
≤T3	9	6		12	4	
T4	4	10	0.139	11	5	1.000
Histological type						
Well, Moderately	10	14		20	7	
Poorly, Mucinous	3	2	0.632	3	2	0.604
The number of lymph node metastases						
0	8	6		14	3	
1-3	3	6		6	3	
≥4	2	4	0.436	3	3	0.292
Preoperative CEA (ng/ml)						
≤5	8	5		11	3	
>5	4	10	0.128	10	6	0.440
Preoperative CA19-9 (U/ml)						
≤37	6	6		8	5	
>37	1	4	0.338	5	1	0.605
The amount of blood lost (ml)						
≤250	9	5		12	4	
>250	4	10	0.128	10	5	0.704
Length of operation (min)						
≤240	11	10		18	6	
>240	2	5	0.396	4	3	0.384
Complication(s)						
No	11	10		18	6	
Yes	2	6	0.238	5	3	0.654
Infectious complication(s)						
No	12	14		21	8	
Yes	1	2	1.000	2	1	1.000
The number of hospitalization days (days)						
≤30	9	4		11	4	
>30	4	12	0.027	12	5	1.000
The number of postoperative days before the initiation of dietary intake (days)						
≤4	10	9		16	5	
>4	3	7	0.433	7	4	0.681
Adjuvant chemotherapy						
No	5	9		10	5	
Yes	8	7	0.462	13	4	0.699

*CI* confidence interval, *CEA* carcinoembryonic antigen, *CA19-9* carbohydrate antigen 19–9, *PNI* prognostic nutritional index

### Prognostic factors influencing the long-term survival in the exploratory study

The correlations between the overall survival and various clinicopathological factors are shown in Table 3. According to a univariate analysis, the overall survival had significant relationships with the combination of pre and postoperative PNI (*p* = 0.013) and the number of lymph node metastases (*p* = 0.008). A multivariate analysis indicated that the combination of pre and postoperative PNI (*p* = 0.030), the number of lymph node metastases (*p* = 0.021) and the adjuvant chemotherapy (*p* = 0.044) were independent risk factors for mortality.

**Table 3:** The correlations between the overall survival and various clinicopathological factors in the exploratory study

	Univariate analysis			Multivariate analysis		
	Hazard Ratio	95 % CI	*p*-value	Hazard Ratio	95 % CI	*p*-value
Age (>70 years)	4.143	0.832–20.641	0.083	0.616	0.054–6.992	0.696
Gender (Female)	0.998	0.238–4.178	0.998			
Location of primary tumor (Rectum)	1.260	0.315–5.040	0.744			
Tumor depth (T4)	1.044	0.261–4.177	0.951	0.098	0.004–2.233	0.145
Histological type (Poorly, Mucinous)	1.929	0.389–9.571	0.422	0.928	0.064–13.370	0.956
The number of lymph node metastases (≥4)	6.656	1.643–26.960	0.008	21.560	1.584–293.440	0.021
Preoperative CEA (>5 ng/ml)	3.218	0.648–15.975	0.153	13.475	0.281–645.979	0.188
Preoperative CA19-9 (>37 U/ml)	1.023	0.187–5.602	0.979			
Adjuvant chemotherapy (None)	1.540	0.368–6.447	0.555	13.021	1.076–157.523	0.044
The amount of blood lost (>250 ml)	1.896	0.453–7.942	0.381			
Combination of pre and postoperative PNI (<45)	3.770	1.316–10.801	0.013	6.728	1.200–37.716	0.030

*CI* confidence interval, *CEA* carcinoembryonic antigen, *CA19-9* carbohydrate antigen 19–9, *PNI* prognostic nutritional index

### Clinical characteristics in the validation study

The patient characteristics are listed in Table 1. The patient population consisted of 100 males and 86 females, with a median age of 67 years (range: 26 to 86). Ninety-four patients had tumors located in the colon and 92 had tumors located in the rectum. Among the total 186 of patients, one hundred and forty-one patients received adjuvant chemotherapy. Among these patients, one hundred and twenty-six patients received monotherapy using an oral pro-drug based on 5-FU, while 15 patients received combination therapy with 5-FU and oxaliplatin.

### Survival analysis according to the pre-/postoperative PNI in the validation study

**Fig. 5:**
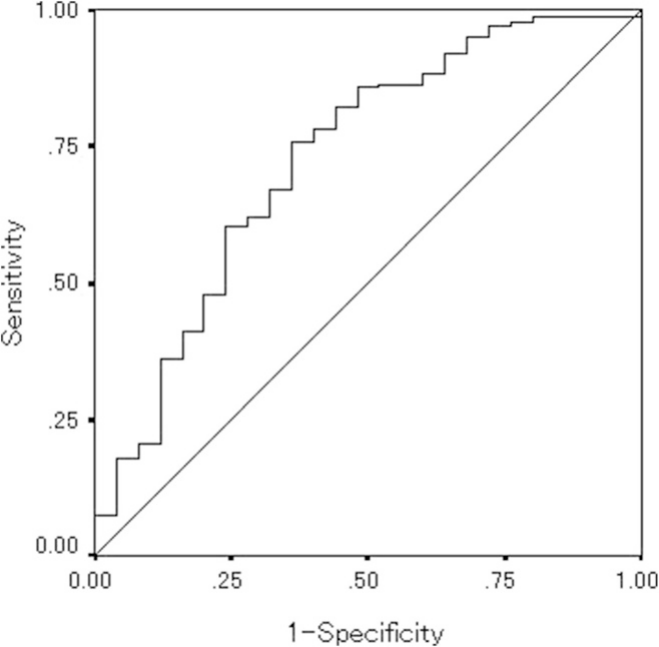
Receiver operating characteristic curve analysis of the postoperative PNI in the validation study. Area under the curve = 0.727, 95 % Confidence interval = 0.612-0.842, *p* < 0.001

**Fig. 6:**
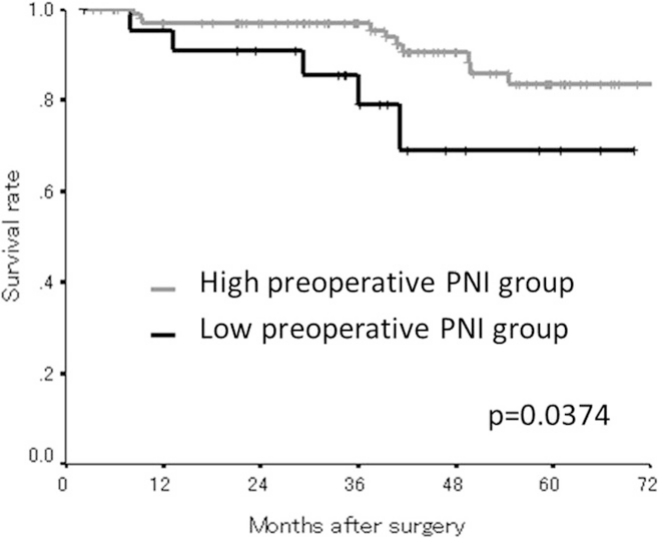
The Kaplan-Meier survival curves according to the preoperative PNI in the validation study. The overall survival rates were significantly worse in the low preoperative PNI group (*p* = 0.0374)

**Fig. 7:**
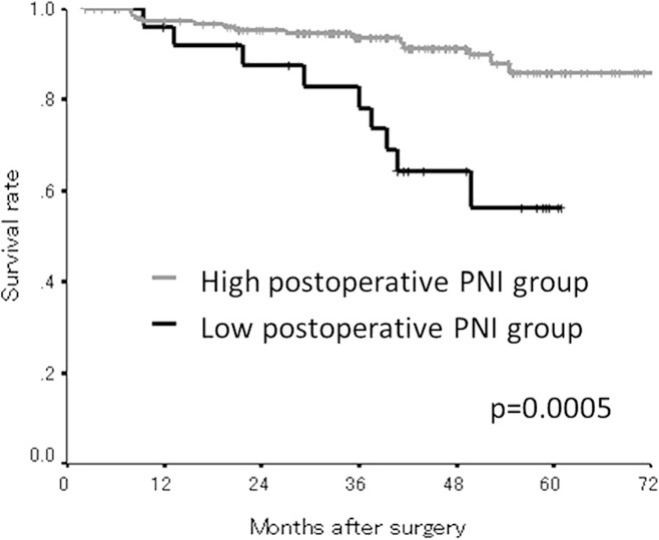
The Kaplan-Meier survival curves according to the postoperative PN in the validation study. The overall survival rates were significantly worse in the low postoperative PNI group (*p* = 0.0005)

The median preoperative PNI was 47.90 (range: 32.45-61.36) and the median postoperative PNI was 48.69 (range: 32.62-66.96) (Table 1). The PNI distribution was normal. According to the Receiver Operating Characteristic (ROC) curve, we set 43.0 as the cut-off value (the sensitivity was 89.4 % and the specificity was 64.0 %) (Fig. 5). Based on the cut-off value of 43.0, 106 patients were classified into the high preoperative PNI group and 23 patients were classified into the low preoperative PNI group. Moreover, 160 patients were classified into the high postoperative PNI group and 26 patients were classified into the low postoperative PNI group. With regard to the preoperative PNI, the overall survival rates were significantly worse in the low PNI group compared to the high PNI group (*p* = 0.0374) (Fig. 6). Moreover, the overall survival rates were also significantly worse in the low postoperative PNI group (*p* = 0.0005) (Fig. 7).

### Survival analysis according to the combination of the preoperative and postoperative PNI in the validation study

**Fig. 8:**
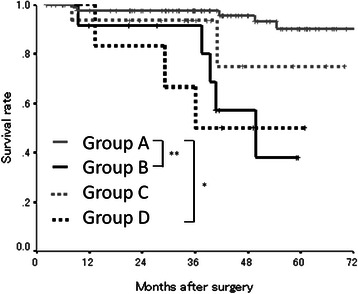
The overall survival subdivided according to the preoperative and postoperative PNI in the validation study. The patients in group A exhibited a better prognosis than those in groups B and D (*, *p* = 0.0001; **, *p* < 0.0001)

We categorized the patients into four groups according to the combination of their preoperative and postoperative PNI in the same manner as the exploratory study. The patients in group A exhibited a better prognosis than the patients in groups B and D (A vs. B, *p* < 0.0001; A vs. D, *p* = 0.0001) (Fig. 8).

### The correlations between the preoperative/postoperative PNI and the clinicopathological factors in the validation study

The preoperative PNI had a significant relationship with gender (*p* = 0.012) and age (*p* = 0.035) (Table 4). The postoperative PNI had a significant relationship with the number of hospitalization days (*p* = 0.035) and the number of postoperative days to initiate dietary intake (*p* = 0.034), and tended to correlate with the preoperative CA19-9 (*p* = 0.088) and the amount of blood loss (*p* = 0.094).

**Table 4:** The relationship between the clinicopathological factors and the preoperative/preoperative PNI in the validation study

	Preoperative PNI			Postoperative PNI		
	High	Low	*p*-value	High	Low	*p*-value
Gender						
Male	52	18		87	13	
Female	54	5	0.012	73	13	0.679
Age (years)						
≤70	65	8		99	16	
>70	41	15	0.035	61	10	1.000
Location of primary tumor						
Colon	57	15		81	13	
Rectum	49	8	0.361	79	13	1.000
Tumor depth						
≤T3	78	15		110	20	
T4	28	8	0.447	50	6	0.493
Histological type						
Well, Moderately	98	22		147	25	
Poorly, Mucinous	8	1	1.000	13	1	0.696
The number of lymph node metastases						
0	42	10		62	5	
1-3	45	10		67	16	
≥4	19	3	0.844	31	5	0.116
Preoperative CEA (ng/ml)						
≤5	74	15		112	14	
>5	22	8	0.286	39	4	1.000
Preoperative CA19-9 (U/ml)						
≤37	89	21		141	14	
>37	4	2	0.340	8	3	0.088
The amount of blood lost (ml)						
≤100	50	11		87	10	
>100	54	12	1.000	67	16	0.094
Length of operation (min)						
≤240	75	13		103	15	
>240	29	10	0.210	51	11	0.379
Complication(s)						
No	78	15		112	17	
Yes	28	8	0.447	47	9	0.647
Infectious complication(s)						
No	84	19		120	22	
Yes	22	4	1.000	39	4	0.453
The number of hospitalization days (days)						
≤17	56	10		92	9	
>17	50	13	0.493	68	17	0.035
The number of postoperative days before the initiation of dietary intake (days)						
≤3	73	18		120	14	
>3	33	5	0.455	40	12	0.034
Adjuvant chemotherapy						
No	78	13		40	5	
Yes	28	10	0.131	120	21	0.628

*CI* confidence interval, *CEA* carcinoembryonic antigen, *CA19-9* carbohydrate antigen 19–9, *PNI* prognostic nutritional index

### Prognostic factors influencing the long-term survival in the validation study

The correlations between the overall survival and various clinicopathological factors are shown in Table 5. According to a univariate analysis, the overall survival had significant relationships with the combination of pre and postoperative PNI (*p* = 0.001), age (*p* = 0.001), histological type (*p* = 0.004), the number of lymph node metastases (*p* < 0.001) and the preoperative CEA level (*p* = 0.033). A multivariate analysis indicated that the combination of pre and postoperative PNI (*p* = 0.001), histological type (*p* = 0.044), the number of lymph node metastases (*p* = 0.022) and the preoperative CEA level (*p* = 0.037) were independent risk factors for mortality.

**Table 5:** The correlations between the overall survival and various clinicopathological factors in the validation study

	Univariate analysis			Multivariate analysis		
	Hazard Ratio	95 % CI	*p*-value	Hazard Ratio	95 % CI	*p*-value
Age (>70 years)	4.070	1.718–9.646	0.001	0.469	0.095–2.313	0.353
Gender (Female)	1.335	0.578–3.085	0.499			
Location of primary tumor (Rectum)	0.492	0.208–1.160	0.105			
Tumor depth (T4)	1.524	0.668–3.480	0.317	0.625	0.163–2.391	0.492
Histological type (Poorly, Mucinous)	4.230	1.567–11.417	0.004	7.723	1.057–56.416	0.044
The number of lymph node metastases (≥4)	4.356	1.920–9.882	<0.001	5.707	1.279–25.465	0.022
Preoperative CEA (>5 ng/ml)	2.709	1.086–6.757	0.033	4.355	1.090–17.394	0.037
Preoperative CA19-9 (>37 U/ml)	1.740	0.401–7.538	0.459			
Adjuvant chemotherapy (None)	0.399	0.093–1.707	0.215	1.110	0.198–6.230	0.906
The amount of blood lost (>100 ml)	0.794	0.335–1.882	0.600	0.271	0.070–1.050	0.059
Combination of pre and postoperative PNI (<43)	2.019	1.327–3.072	0.001	2.542	1.490–4.337	0.001

*CI* confidence interval, *CEA* carcinoembryonic antigen, *CA19-9* carbohydrate antigen 19–9, *PNI* prognostic nutritional index

## Discussion

In this study, we investigated the correlations between the postoperative PNI and the long-term outcome in patients with stage II/III CRC. To the best of our knowledge, this is the first study to evaluate the prognostic significance of the postoperative PNI.

The preoperative PNI, which reflects the nutritional and immunological status of the host and was initially used as a predictor of complications after digestive surgery, was reported to correlate with the long-term outcome in patients with esophageal [5], gastric [6], pancreatic [7] and colorectal cancer [8, 9]. Although the prognostic significance of the preoperative PNI has been reported, there have been few reports which have focused on the prognostic significance of the postoperative PNI. Based on the results of the present study, the postoperative status was also considered to correlate with the long-term outcome. Moreover, the combination of the preoperative and postoperative PNI enabled a more accurate stratification of the risk for a poor prognosis.

The PNI can be easily calculated from the serum albumin concentration and the lymphocyte count, which are standard parameters assessed in the clinical setting. Although the details of the relationship between a low PNI and a poor prognosis are currently unclear, the mechanism(s) is considered to include the following: Hypoalbuminemia reflects malnutrition in the patient [11], and malnutrition has been reported to correlate with an immunosuppressed condition [12, 13]. In some previous reports, hypoalbuminemia itself was reported to be a prognostic factor for a poor survival in patients with malignancies [14–16]. Moreover, lymphopenia was also previously reported to be a prognostic factor for poor survival in patients with malignant disease [17, 18]. The low lymphocyte count is associated with a preexisting immunosuppressed condition, suggesting that the host has an inadequate anti-tumor immunological reaction [19, 20]. Therefore, lymphopenia creates a favorable microenvironment for recurrence [21]. Taken together, the low postoperative PNI, which consists of the serum albumin concentration and lymphocyte count, correlates with the survival.

Although attention has been focused on the preoperative status of the host in previous reports, the present study indicates that the long-term outcome should be considered to correlate with the postoperative status, as well as the preoperative status.

In this study, the postoperative PNI tended to correlate with the amount of blood lost during the operation. Mörner et al. previously reported that the degree of intraoperative blood loss was a factor that influenced the long-term survival [22]. In this study, the amount of blood lost was associated with the postoperative PNI, and a low postoperative PNI was associated with poor survival. These results support the theory that increasing the amount of intraoperative blood loss could have a negative impact on the prognosis.

Although it was expected that the postoperative complications which lead to hypoalbuminemia caused by the systemic inflammatory response or long-term fasting might correlate with the low postoperative PNI, there was no relationship found between the postoperative complications and postoperative PNI in this study. However, the grade of the postoperative complications was not taken into consideration in this study. Therefore, it cannot be concluded that the postoperative complications and the postoperative PNI are unrelated. Due to the correlation that was observed in this study between a low postoperative PNI and an extended number of hospitalization days and a delay in initiating dietary intake, some relationships might exist between a low postoperative PNI and the lack of a favorable recovery.

Although it has been suggested that the PNI tends to correlate with age [23, 24] and the preoperative PNI correlated with age in this study, the correlation between the postoperative PNI and age was not seen in this study. This may be because various postoperative factors, which were not identified in this study, exceed the impact of age in terms of the effect on the postoperative PNI.

There are some possible limitations associated with this study. First, we evaluated a relatively small number of patients. Moreover, this study was a retrospective study, and the criteria for the first visit after leaving the hospital were not uniform. Therefore, the criteria regarding the timing of measuring the PNI was also not uniform. A large, prospective study should therefore be performed to confirm our findings.

## Conclusions

In this study, the postoperative PNI was demonstrated to be a useful predictor of a poor prognosis in patients with CRC. This result confirmed that the postoperative nutritional and immunological status are important when considering the long-term outcome.

## Abbreviations

CRC: Colorectal cancer
PNI: Preoperative prognostic nutritional index
ROC: Receiver operating characteristic
CEA: Carcinoembryonic antigen
CA19-9: Carbohydrate antigen 19–9
